# Modelling the Ecological Comorbidity of Acute Respiratory Infection, Diarrhoea and Stunting among Children Under the Age of 5 Years in Somalia

**DOI:** 10.1111/insr.12206

**Published:** 2017-01-26

**Authors:** Damaris K. Kinyoki, Samuel O. Manda, Grainne M. Moloney, Elijah O. Odundo, James A. Berkley, Abdisalan M. Noor, Ngianga‐Bakwin Kandala

**Affiliations:** ^1^ Spatial Health Metrics Group, INFORM ProjectKenya Medical Research Institute/Wellcome Trust Research ProgrammeNairobiKenya; ^2^ Biostatistics Research UnitSouth African Medical Research CouncilPretoriaSouth Africa; ^3^ Division of Epidemiology and Biostatistics, School of Public HealthUniversity of WitwatersrandJohannesburgSouth Africa; ^4^ Nutrition Section, United Nations Children's Fund (UNICEF)Kenya Country Office, UN Complex GigiriNairobiKenya; ^5^ Food Security and Nutrition Analysis Unit (FSNAU) for SomaliaUnited Nations Food and Agricultural OrganizationNgecha Road CampusNairobiKenya; ^6^ Kenyan Medical Research Institute/Wellcome Trust Research ProgrammeCentre for Geographic Medicine Research (Coast)KilifiKenya; ^7^ Centre for Tropical Medicine and Global Health, Nuffield Department of Clinical MedicineUniversity of Oxford, CCVTMOxfordOX3 7LJUK; ^8^ Warwick Evidence, Warwick Medical SchoolUniversity of WarwickGibbet HillCoventryCV4 7ALUK; ^9^ Department of Mathematics and Information Sciences, Faculty of Engineering and EnvironmentNorthumbria UniversityNewcastle upon TyneNE1 8STUK

**Keywords:** Diarrhoea, acute respiratory infections, stunting, geostatistics, shared component, Somalia

## Abstract

The aim of this study was to assess spatial co‐occurrence of acute respiratory infections (ARI), diarrhoea and stunting among children of the age between 6 and 59 months in Somalia. Data were obtained from routine biannual nutrition surveys conducted by the Food and Agriculture Organization 2007–2010. A Bayesian hierarchical geostatistical shared component model was fitted to the residual spatial components of the three health conditions. Risk maps of the common spatial effects at 1×1 km resolution were derived. The empirical correlations of the enumeration area proportion were 0.37, 0.63 and 0.66 for ARI and stunting, diarrhoea and stunting and ARI and diarrhoea, respectively. Spatially, the posterior residual effects ranged 0.03–20.98, 0.16–6.37 and 0.08–9.66 for shared component between ARI and stunting, diarrhoea and stunting and ARI and diarrhoea, respectively. The analysis showed clearly that the spatial shared component between ARI, diarrhoea and stunting was higher in the southern part of the country. Interventions aimed at controlling and mitigating the adverse effects of these three childhood health conditions should focus on their common putative risk factors, particularly in the South in Somalia.

## 1 Introduction

Illness in children living in developing countries is commonly characterised by more than one health condition (Fenn, Morris, & Black, 2005). Diarrhoea and respiratory infections, which both impair growth, remain the leading cause of death and often co‐occur in children under the age of 5 years (Black, Morris, & Bryce, 2003; Torres *et al*., 2000). The comorbidity of the two health conditions may be as a result of shared risk factors at child level, or as a result of shared extrinsic factors. For instance, diarrhoea and respiratory infection may both share age as a child‐dependent risk factor or poor sanitation and crowding as the environmental risk factors (Kosek, Bern, & Guerrant, 2003; Williams, Gouws, Boschi‐Pinto, Bryce, & Dye, 2002).

The relationship between nutritional status and infection, specifically diarrhoea and respiratory diseases has been studied widely (Black, 1991; Guerrant, Schorling, McAuliffe, & de Souza, 1992). Studies show that height deficits are proportional to diarrheal prevalence (Checkley, Epstein, Gilman, Cabrera, & Black, 2003). However, children can demonstrate catch‐up growth with adequate nutrition and improved environmental conditions and sufficient time between diarrhoea episodes (Briend, Hasan, Aziz, & Hoque, 1989; Golden, 1994). Persistent or frequent diarrhoeal diseases in children can lead to permanent growth retardation (Lutter et al., 1989; Rowland, Rowland, & Cole, 1988; Victora, Barros, Kirkwood, & Vaughan, 1990). Persistent diarrhoea in children is often associated with respiratory tract infections (Fenn *et al*., 2005; Schmidt, Cairncross, Barreto, Clasen, & Genser, 2009). Additionally, malnutrition or linear growth retardation are known risk factors of respiratory tract infections (Caulfield, de Onis, Blossner, & Black, 2004; Savitha, Nandeeshwara, Pradeep Kumar, ul‐Haque, & Raju, 2007).

In Somalia, a high level of conflict has been cited as the main impediment to accessing health care and humanitarian aid (UN‐FAO/FSNAU‐Somalia, 2011; WHO, UNICEF, WFP, & FAO/FSNAU‐Somalia, 2010). The common causes of morbidity and mortality in Somalia are diarrhoea, including cholera, respiratory infection, malaria and measles (Laughlin & Legters, 1993; Oldfield, Rodier, & Gray, 1993). In the South Central zone, where there are often high levels of conflict, malnutrition in children below the age of 5 years is reported to be at critical levels (Kinyoki, Berkley, *et al*., 2016; Kinyoki, Kandala, *et al*., 2016). In this zone, settlement patterns in vital urban and agricultural areas have undergone substantial changes, with large influxes of non‐resident clans, supported by their militias for economic gains. This has disrupted agro‐pastoral livelihood, which translates into food insecurity among the affected communities (UNFAO, 1997). As a result of these insecurities, Somalia has a large number of internally displaced people. Internally displaced people are considered to be among the poorest and vulnerable to infections (FAO/FSNAU‐Somalia, 2011a). Limited access because of insecurity in the South Central zone in Somalia has inhibited health activities, contributing to spread and sustained high levels of these diseases (Seal & Bailey, 2013). In addition, because of internal displacement and drought, food insecurity and health problems, the country continues to suffer high malnutrition rates (Seal & Bailey, 2013). The co‐occurrence of these conditions predisposes the population to high levels of morbidity and mortality (Laughlin & Legters, 1993).

Estimating comorbidity of childhood diseases informs the ranking of different public health interventions in terms of potential combined effects (Mulholland, 2005). While there is evidence of the co‐occurrence of diseases at the child level, studies that have looked at ecological associations between the childhood illnesses are sparse in the context of the Sub‐Saharan African region. A study by Kazembe and Namangale in 2007 combined a set of childhood illness into a multinomial outcome and used univariate spatial methods on specific categories of the derived outcomes. In the present study, we model the comorbidity of acute respiratory infections (ARIs), diarrhoea and stunting among children of the age of 6–59 months in Somalia in order to identify areas where comorbidity is most prevalent. We model each outcome within a child separately but assess the common spatial patterns by imposing joint effects on their residual spatial effects. Additionally, we assess geographical comorbidity in children at very finer resolution, which provides a better picture of risk gradient in space. We used a shared‐component model to fit common unobserved and unmeasured spatial risks (Held, Graziano, Frank, & Rue, 2006; Kandala, Manda, Tigbe, Mwambi, & Stranges, 2013; Manda, Feltbower, & Gilthorpe, 2011).

## 2 Methods

### 2.1 Survey and Data

#### 2.1.1 Surveys

We obtained data for this study from the Food Security and Nutrition Unit (FSNAU). The FSNAU mandate is to provide evidence‐based analysis of Somali food, nutrition and livelihood security to enable both short‐term emergency responses and long‐term strategic planning. Therefore, in partnership with United Nations Children's Fund, FSNAU has been conducting biannual seasonal nutrition assessment surveys since 2001. Our study utilised survey data ranging from the year 2007 to 2010 (FSNAU 2007–2010) (FAO/FSNAU‐Somalia, 2011b; WHO, UNICEF, WFP & FAO/FSNAU, 2010). A stratified, multi‐stage cluster sampling design was used where the sampling frame of a selected district is based on three livelihood definitions (pastoral, agro‐pastoral and riverine), within which 30 rural communities and 30 households within each community were selected at random (Noor *et al.*, 2008). A list of all villages and populations within each of the assessed livelihoods was used to construct cumulative population for the assessment area. The selection of respondents within the village was carried out randomly, from a list of eligible names or a map of households. Therefore, the data for this study were hierarchical in nature, where the children were nested in their respective households and households in the clusters. Details of the survey methods and data collection are described elsewhere (FAO/FSNAU‐Somalia, 2011b).

#### 2.1.2 Data

At the individual child level, information collected was on child age, gender, weight, height, mid‐upper arm circumference, vitamin A supplementation in the last 6 months, diarrhoea, ARI and febrile illness in the 2 weeks before the survey and polio and measles vaccination history. At the household level, information recorded included the household size and age structure, gender of the household head and access to different types of foods in the last 24 h. *Z*‐scores were computed for stunting using the World Health Organization 2006 references (De Onis & Blossner, 2003). Stunting was defined as below −2*Z*‐score for height for age.

The effect of a set of four distal environmental covariates associated with food security (Grace, Davenport, Funk, & Lerner, 2012) and vector‐borne diseases (Noor *et al.*, 2014) on indices of malnutrition was examined. These were rainfall, enhanced vegetation index (EVI), mean temperature and urbanisation. Rainfall and mean temperature were derived from the monthly average grid surfaces obtained from WorldClim database. The EVI values were derived from the moderate‐resolution imaging spectroradiometer sensor imagery (Scharlemann *et al.*, 2008) for the period 2000–2010, while the urbanisation information was obtained from Global Rural–Urban Mapping Project (Balk *et al.*, 2005). All environmental covariates were extracted from 1 × 1 km spatial resolution grids. Rainfall, temperature and EVI were summarised to compute seasonal averages using the four main seasons in Somalia: December to March, a harsh dry season called ‘Jilal’; April to June, which is the main rainy season called ‘Gu’; from July to September is the second dry season, the ‘Hagaa’; and October to December, the short rainy season known as ‘Deyr’.

### 2.2 Statistical Methods

Our main analysis used a joint modelling for multiple conditions approach (Held *et al*., 2006; Held, Natario, Fenton, Rue, & Becker, 2005; Manda *et al*., 2011) to investigate comorbidity of ARI, diarrhoea and stunting at child level using shared components in integrated nested Laplace approximation (INLA) as implemented in the r‐inla library (Lindgren & Rue, 2015). This was carried out controlling for child, household and community level factors that might influence the relationship. Specifically, we let *y*_*ijk*_ be a binary response variable where *y*_*ijk*_=1 if a child *i* at cluster*j*with condition *k* and *y*_*ijk*_=0 if a child did not have any outcome of interest where *i*=1,…,73778, *j*=1,…,1066 and *k*=1,2,3. The response variables were distributed as Bernoulli random variables such that

$$f(y_{ijk}|\eta_{ijk})=p_{ijk}^{y_{ijk}}{\left(1-p_{ijk}\right)}^{1-y_{ijk}}=\exp[y_{ijk}\eta_{ijk}-log(1+\exp(\eta_{ijk}))]$$

where *p*_*ijk*_=*P*(*y*_*ijk*_=1), and $n_{ijk}=logit(p_{ijk})$ were canonical parameters linked to the linear predictor. The multivariate spatial effects for ARI *u*_1_, diarrhoea *u*_2_ and stunting *u*_3_ were defined through a coregionalisation model (Krainski, Lindgren, Simpson, & Rue, 2016; Schmidt & Gelfand, 2003) given by

$$\begin{array}{ccc} u_{1}(s) & =z_{1}(s) & \\ u_{2}(s) & =\lambda_{1}*z_{1}(s)+z_{2}(s)\\ u_{3}(s) & =\lambda_{2}*z_{1}(s)+\lambda_{3}*z_{2}(s)+z_{3}(s) \end{array}$$

where *z*_1_,*z*_2_,*z*_3_ were Matérn spatial fields that described the shared component of the health conditions. The binary classification of child *i* in cluster *j* for disease *k* was modelled by child cluster‐specific covariates, disease‐specific intercept and spatial field as

$$\begin{array}{ccc} y_{ijk} & ∼\text{Bernoulli}(p_{ijk}) & \\ p_{ijk} & ={\text{logit}}^{-1}\eta_{ijk}=\alpha_{k}+x_{ij}\beta_{k}+u_{k}(s_{j}) \end{array}$$

where *β*_*k*_ were the disease‐specific risk coefficients associated with the risk vector $x^{\prime }$ of ARI, diarrhoea and stunting. In this approach, the univariate Matérn spatial fields were implemented through the stochastic partial differential equation (SPDE) approach as implemented in r‐inla (Rue, Martino, & Chopin, 2009). This SPDE is formulated as a link between Gaussian random fields and the Gaussian Markov random fields (Lindgren & Rue, 2015). The covariance function and the dense covariance matrix of the Gaussian field are replaced by a neighbourhood structure and a sparse precision matrix, respectively, that together define a Gaussian Markov random field (Cameletti, Lindgren, Simpson, & Rue, 2013).

$$x(u)=\overset{n}{\underset{i=1}{\sum }}\psi_{i}(u)w_{i}$$

Here, the $\left\{w_{i}\right\}$ represents the Gaussian distributed weights and *ψ*_*i*_ are piecewise linear basis functions defined on a triangulation of the domain with *n* nodes defined as mesh. We used a non‐stationary model by modifying the SPDE to obtain the Gaussian random fields with defined dependence structure expressed as

$$(k(u^{2})-\Delta )(\tau (u)x(u))=w(u),u\in \Omega^{2},$$

We used default priors for the fields as defined in Lindgren and Rue, 2015, and penalised complexity priors for the copy parameter (Simspon *et al.*, 2015). Further, the marginal excursion probabilities based on the estimated posterior distribution of the shared component were simultaneously calculated using the quintile correction method as implemented by Bolin and Lindgren, 2015. Marginal probabilities where the shared component exceeded 0.2 level were determined using the excursion function in the quintile correction method and the parametric family of excursion sets (Bolin & Lindgren, 2015). To assess the model performance, we compared the deviance information criteria, Watanabe–Akaike information criterion and conditional predictive ordinate (Gelman, Hwang, & Vehtari, 2014) for the main shared component model and separate models for stunting, diarrhoea and ARI (Cameletti, Lindgren, Simpson, & Rue, 2013).

### 2.3 Ethical Approval

Ethical approval was provided by the Ministry of Health Somalia, Transitional Federal Government of Somalia Republic, Ref: MOH/WC/XA/146./07, dated 02/02/07. Because of the high illiteracy rate of the population, informed verbal consent was sought from all participating households and individuals. An additional 10% was added to the sample size for the surveys to allow for dropout or refusal to participate.

## 3 Results

**Figure 1:**
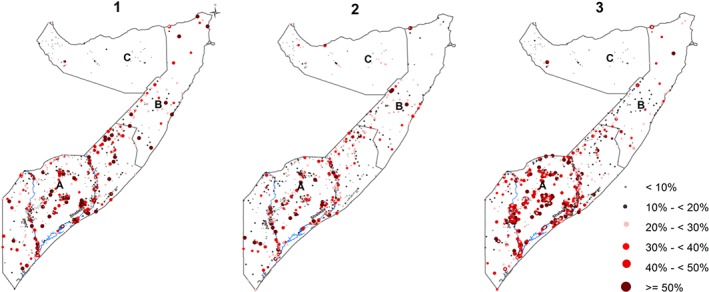
Maps showing the crude prevalence of acute respiratory infection (ARI), diarrhoea, stunting by clusters for Food Security and Nutrition Unit nutrition surveys conducted between 2007 and 2010 in Somalia. The country is divided into three main zones: (A) South Central, (B) North West, (C) North East. Map 1, ARI; 2, diarrhoea; 3, stunting. The country's two main rivers, Juba and Shebelle, are located in the South Central zone. [Colour figure can be viewed at wileyonlinelibrary.com]

Information from 1 066 unique survey locations with a total of 73 778 children were used in the analysis. Figure 1 shows the spatial distribution of the clusters. The location of 1 765 children from 34 clusters could not be accurately determined and were therefore excluded from the analysis. Of the 73 778 children included, 12 641 (17*%*) had ARI in the last 1 week, 18 939 (26*%*) had diarrhoea in the last 1 week and 22 739 (31%) were stunted (Table 1 and Table SI.1). The correlation of environmental covariates was found to be low and therefore were all included in the joint modelling (Figure SI.1). The test of association between the three conditions were all significant using the chi‐squared test (Table 2).

**Table 1:** The summary of the data used in this study, summarised by zone and region.

			Number of children	Number with	Number with	Number
Zone	Region	Cluster	examined	diarrhoea	ARI	stunting
North East (Puntland)	Bari	9	756	535	497	201
	Mudug	61	6188	783	765	804
	Nugaal	24	1673	384	422	383
North West (Somaliland)	Awdal	26	862	51	58	7
	Sanaag	14	412	23	54	3
	Sool	3	142	25	48	18
	Togdheer	12	673	435	849	362
	Woqooyi Galbeed	23	2465	768	1128	1378
South Central	Bakool	75	3534	543	1087	1150
	Banadir	1	51	39	14	0
	Bay	98	5568	1236	1876	2133
	Galgaduud	77	5831	1342	1710	1908
	Gedo	111	6985	1048	1851	1999
	Hiraan	142	10743	947	1578	2260
	Juba Dhexe	77	5253	967	1463	2734
	Juba Hoose	71	5560	1514	1617	1553
	Shabelle Dhexe	101	7650	878	1587	2414
	Shabelle Hoose	141	9432	1123	2335	3432
Grand total	18	1066	73778	12641	18939	22739

The table gives the number of sampled unique clusters in every region, the number of children examined and the number and percentage of children who were stunted.

ARI, acute respiratory infection.

**Table 2:** Relationship of acute respiratory infection (ARI), diarrhoea and stunting using chi‐squared test.

	Degree of freedom	Deviance	Residual degree of freedom	Residual deviance	*P* value
Stunting/diarrhoea	1	183.04	73771	74218	<0.001
Stunting/ARI	1	15.74	73771	96769	<0.001
ARI/diarrhoea	1	2081.61	73771	72319	<0.001

The correlates of the three health conditions from the shared component model are shown in Table SI.2. Increases in the household size and number of under‐fives in the household were associated with increased risk of stunting, diarrhoea and ARI. Children who had consumed any of the staple sources of carbohydrates or proteins within the 24 h prior to the survey had a lower risk of all the three health conditions. Some of the environmental factors that were associated with lower risk of the stunting, diarrhoea and ARI were vegetation cover, rainfall and urbanisation.

**Figure 2:**
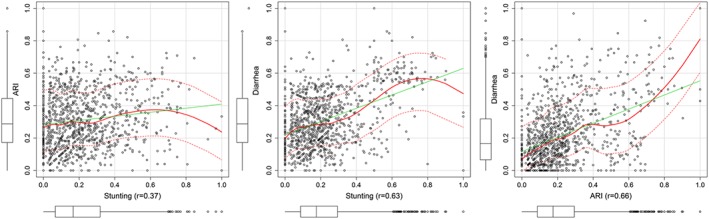
Correlation plots of acute respiratory infection (ARI), diarrhoea and stunting among children under the age of 5 years in Somalia. [Colour figure can be viewed at wileyonlinelibrary.com]

As a first step towards joint modelling, empirical correlations of the proportion of children with the three conditions at cluster level were determined (Figure 2). All of the conditions showed a positive correlation. ARI and diarrhoea had the highest correlation at 0.66, followed by diarrhoea and stunting at 0.63, and the lowest correlation was between ARI and stunting at 0.37.

**Figure 3:**
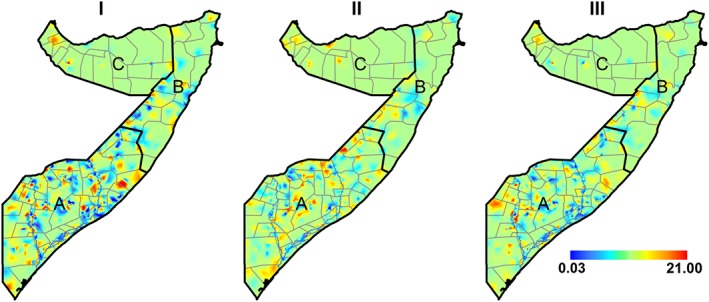
Posterior residual shared spatial effects common to (I) stunting and acute respiratory infection (ARI); (II) stunting and diarrhoea; (III) diarrhoea and ARI. A, South Central zone; B, North East (Puntland) zone; C, North West (Somaliland) zone. [Colour figure can be viewed at wileyonlinelibrary.com]

The shared spatial residual effects were significant (OR = 1.03, 95*%* CrI: 1.01–1.05); (OR = 1.36, 95*%* CrI: 1.15–1.60); and (OR = 1.55, 95*%* CrI: 1.44–1.66) for ARI and stunting; diarrhoea and stunting; and diarrhoea and ARI, respectively. Estimated shared spatial residual effects are shown in Figure 3. The shared spatial effects for ARI and stunting were higher in the South and lower in the North, while shared spatial effects for diarrhoea and stunting and ARI and diarrhoea were higher in the South Central and the North East and low in the North West of Somalia. All the three components were high along the two main rivers in Somalia: Juba and Shebelle as compared with the northern region of Somalia. The districts that were most affected were in the South Central zone. The posterior correlation means ranged 0.03–20.98, 0.16–6.37 and 0.08–9.66 for shared component between ARI and stunting, diarrhoea and stunting and ARI and diarrhoea, respectively. The distribution of shared component had a larger effect on stunting and diarrhoea prevalence in the south.

**Figure 4:**
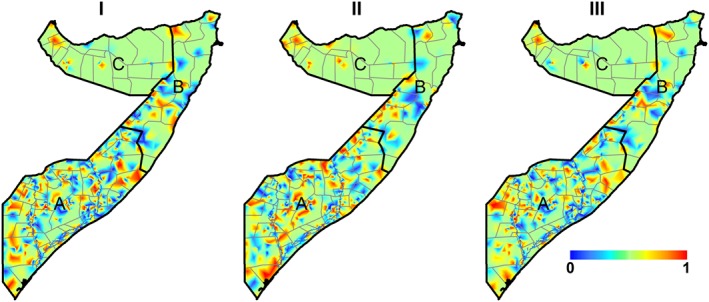
Marginal probability of the following spatial fields: (I) stunting and acute respiratory infection (ARI); (II) stunting and diarrhoea; (III) diarrhoea and ARI. A, South Central zone; B, North East (Puntland) zone; C, North West (Somaliland) zone. [Colour figure can be viewed at wileyonlinelibrary.com]

The maps of marginal excursion probabilities for the level 0.2 based on the estimated posterior distribution are shown in Figure 4. The areas with high marginal probabilities exceeding 0.2 are mainly in the south for all the spatial effects. Maps of standard deviation and summary of the model parameters are in Figures SI.2 and SI.3. The main joint model performed better compared with separate models without the shared field for stunting, diarrhoea and ARI (Table 3).

**Table 3:** Four performance indices to evaluate the predictive performance and model fit for the joint and separate models for stunting, diarrhoea and ARI.

	Joint model				Separate models			
	DIC	WAIC	CPO	PIT	DIC	WAIC	CPO	PIT
Stunting	6 993.62	7 296.42	73.14	546.43	7 370.77	7 692.59	68.65	522.93
Diarrhoea	6 484.78	6 832.56	93.30	521.39	7 103.62	7 535.37	7 535.37	7 535.37
ARI	7 311.19	7 823.22	72.43	479.91	7 696.57	8 272.90	63.66	449.93

ARI, acute respiratory infection; DIC, deviance information criteria; WAIC, Watanabe–Akaike information criterion; CPO, conditional predictive ordinate; PIT, probability integral transform.

## 4 Discussion

In this work, we used a shared component model to jointly analyse three health conditions: ARI, diarrhoea and stunting. The statistical framework used in this study has an advantage in that its latent component has a direct interpretation in terms of the prevalence of comorbidities and related risk factors, which are either shared by several or specific to one of the health conditions. We have demonstrated significant co‐occurrence of ARI, diarrhoea and stunting among children below the age of 5 years in Somalia. Although the mean posterior spatial effects were highest between the ARI and diarrhoea, there were more hot spots for the co‐incidence of diarrhoea and stunting and between ARI and stunting.

Our findings are consistent with previous studies that suggest that both the cumulative incidence and longitudinal prevalence of diarrhoea have a significant association with stunting prevalence (Checkley *et al.*, 2008). In childhood, diarrhoea contributes to malnutrition by impairing weight gain over a short period, while significant impairment in height gain occurs over a longer period (Briend, Hasan, Aziz, & Hoque, 1989). It is also thought that a child needs to recover from weight loss before resuming linear growth, and this contributes to reduction in catch‐up growth (Richard *et al.*, 2014). Recurrent weight faltering associated with multiple diarrhoea episodes may lead to linear growth faltering, but catch‐up growth is possible given adequate diet and time between infections (Richard *et al.*, 2014). However in resource‐poor settings such as Somalia, where dietary intake is consistently inadequate and there are high rates of infectious diseases, the process of catch‐up growth may never be possible resulting in a high level of stunting (Richard *et al.*, 2012; Richard *et al.*, 2014). A biological link between diarrhoeal diseases and ARI is plausible where persistent diarrhoeal diseases may lead to acute malnutrition, which in turn increases the risk of ARI (Schmidt, Cairncross, Barreto, Clasen, & Genser, 2009).

Somalia is highly prone to drought and famine. As a result, malnutrition is the main cause of morbidity and mortality in the country, although imprecisely recorded (FAO/FSNAU‐Somalia, 2010). Persistent conflict has also exacerbated the situation by increasing the number of internally displaced people. Crowding, lack of human waste disposal systems and treated public water supplies in displaced persons centres and feeding stations have degenerated living conditions, hence contributing to the spread of diarrhoeal diseases and respiratory infections (FAO/FSNAU‐Somalia, 2011a; Laughlin & Legters, 1993). A high population of internally displaced people is found in the South Central zone, reflecting the frequent hot spots of the shared components in the region (FAO/FSNAU‐Somalia, 2011b).

It is important to understand the prevalence of the co‐occurrence of health conditions at community level in order to formulate prevention strategies common to the conditions (Mulholland, 2005). Despite the underlying and proximate causes of the conditions, ignoring comorbidity in this population where the prevalence is high could lead to inappropriate prioritisation of public health interventions for reducing the under‐five mortality (Fenn, Morris, & Black, 2005). Our study not only estimates the prevalence of the shared components between the health conditions but also gives the spatial distribution of the components pointing out the priority regions with hot spots where interventions should be targeted. In addition, there were several common underlying correlates associated with the three health conditions that influenced the spatial co‐distribution in Somalia. Access to foods high in protein and carbohydrates and vegetation cover, a proxy of rainfall or drought, were strong correlates of these health conditions. Age, gender, illness, access to carbohydrates and temperature were also common correlates. The correlates and the extent of geographical co‐occurrence of stunting, diarrhoea and ARI can be used to develop more informed interventions to achieve maximum impact within the short term and available funding in Somalia.

There are several limitations to this study. Our modelling approach assumes that the latent risk factors have spatial structure. In addition, our methods assume that the components are independent, hence, no interaction between the effects of unknown covariates (Held, Natario, Fenton, Rue, & Becker, 2005). The prevalence of the comorbidity of diseases is highly dependent on the population characteristics (Fenn *et al.*, 2005), and therefore, the results from this study cannot be generalised to other populations. In addition, the data used in this study were from cross‐sectional studies, and therefore, we are not able to account for the entire history of the disease exposure or reverse causality of ARI, diarrhoea and stunting (Checkley *et al.*, 2008). For this reason, further research should be undertaken using longitudinal studies to better understand the relationship between the health conditions and help define the role of control programmes in the prevention of these health conditions (Checkley *et al.*, 2008).

## 5 Conclusion

In general, a shared component model demonstrates that comorbidity between diarrhoea and stunting and ARI and diarrhoea are high, while the comorbidity between ARI and stunting is relatively low. Significant hot spots of these health conditions have been highlighted in the South Central zone of the country. Therefore, our evidence supports integrated programming and interventions. Intervention aimed at reducing the rates of comorbidity should focus on the hot spots particularly in the South Central region in Somalia. In addition, the model used is able to identify common spatial patterns of more than one health condition, which provides direct guidance for interventions.

## Contributors

D. K. K., N.‐B. K., S. O. M., A. M. N. and J. A. B. were responsible for the concept and design of the study. D. K. K. led the development of the model, data assembly process, data analysis and interpretation of results. G. M. M. and E. O. O. were responsible for conducting the surveys, cleaning and archiving the data. A. M. N., N.‐B. K., S. O. M. and J. A. B. were responsible for overall scientific oversight. All authors reviewed the manuscripts and contributed to the final submission.

## Competing Interests

None declared

## Supporting information

Supporting info item
