# Supplementary material for: Modelling the Ecological Comorbidity of Acute Respiratory Infection, Diarrhoea and Stunting among Children Under the Age of 5 Years in Somalia
Source: Int Stat Rev. 2017 Jan 26;85(1):164–76. doi: 10.1111/insr.12206 (PMC5396332; doi:10.1111/insr.12206)
Supplement: Supplementary file 1 — Supporting info item [file INSR-85-164-s001.docx]

**Supplementary Information: Data description and model output**

**SI.1 Data description**

A set of five geographical covariates were examined; rainfall, enhanced vegetation index (EVI), temperature and urbanization. Rainfall and temperature were derived from the monthly average rasters obtained from WorldClim datasets and were summarized to get the mean annual rainfall and mean annual temperature (1). The EVI values were derived from the MODerate-resolution Imaging Spectroradiometer (MODIS) sensor imagery (2) while the urbanization was obtained from Global Rural Urban Mapping Project (GRUMP) (3). Table SI.1 gives model output of the covariates used in the study.

**Table SI.1:** This table gives detailed information of the variables that were used in the study. Three response variables were defined namely; wasting, stunting and MUAC-for-age using WHO standard population and cut-off points. The predictors were divided into three categories; child specific predictors, household level predictors which also included food and nutrition predictors, and climatic or environmental variables.

| **Variable** | **Type** | **Description** |
| --- | --- | --- |
| **Response variables** | | |
| Diarrhoea | Categorical | 1 = diarrhoea (In the last 2 weeks)  0 = otherwise |
| Acute Respiratory Infection (ARI) | Categorical | 1 = ARI positive (In the last 2 weeks)  0 = otherwise |
| Stunting | Categorical | 1 = stunted (cut-off point <-2 SD = malnutrition)  0 = otherwise |
| **Child specific predictors** | | |
| Vitamin supplements | Categorical | 1 = yes (In the last 6 months)  0 = otherwise |
| Measles vaccinations | Categorical | 1 = yes  0 = otherwise |
| Polio vaccination | Categorical | 1 = yes (Complete doses)  0 = otherwise |
| Febrile Illness | Categorical | 1 = fever positive (in the last 2 weeks)  0 = otherwise |
| Suspected measles | Categorical | 1 = yes (In last 1 month)  0 = otherwise |
| Gender | Categorical | 1 = Female  0 = otherwise |
| Age of the child | Continuous | Age of children from 6 to 59 months (in months) |
| **Household level predictors** | | |
| Household size | Continuous | Number of people in the household |
| Number of under fives | Continuous | Number of children under the age of five years in the household (0-59 months) |
| Household gender female | Categorical | 1= Female  0 = Male |
| Age of the mother | Continuous | Age of the mother in years |
| MUAC of the mother | Continuous | MUAC of the mother in cm |
| **Food and nutrition predictors** | | |
| Carbohydrates | Categorical | 1 = yes (Access to at least one type of carbohydrates in the last 24 hours)  0 = otherwise |
| Protein | Categorical | 1 = yes (Access to at least one type of protein in the last 24 hours)  0 = otherwise |
| Fats | Categorical | 1 = yes (Access to at least one type of Fat in the last 24 hours)  0 = otherwise |
| Fruits and vegetables | Categorical | 1 = yes (Access to at least one type of Fruits and vegetables in the last 24 hours)  0 = otherwise |
| **Climatic / Environmental data** |  |  |
| Enhanced Vegetation Index (EVI) | Continuous | Ranges from 0-1 and derived from temporal Fourier analysed Advanced Very High Resolution Radiometer (AVHRR) data |
| Rainfall | Continuous | Seasonal mean rainfall in mm obtained from WorldClim dataset |
| Temperature | Continuous | Annual mean temperature in ˚C obtained from WorldClim dataset |
| Urbanization | Categorical | Global Rural Urban Mapping Project Modified (GRUMPMod), 1=Urban, 0=Rural |
| Season | Categorical | Jilal (December to March); Gu (April to June); Hagaa (July to September); Deyr (October to December) |

**
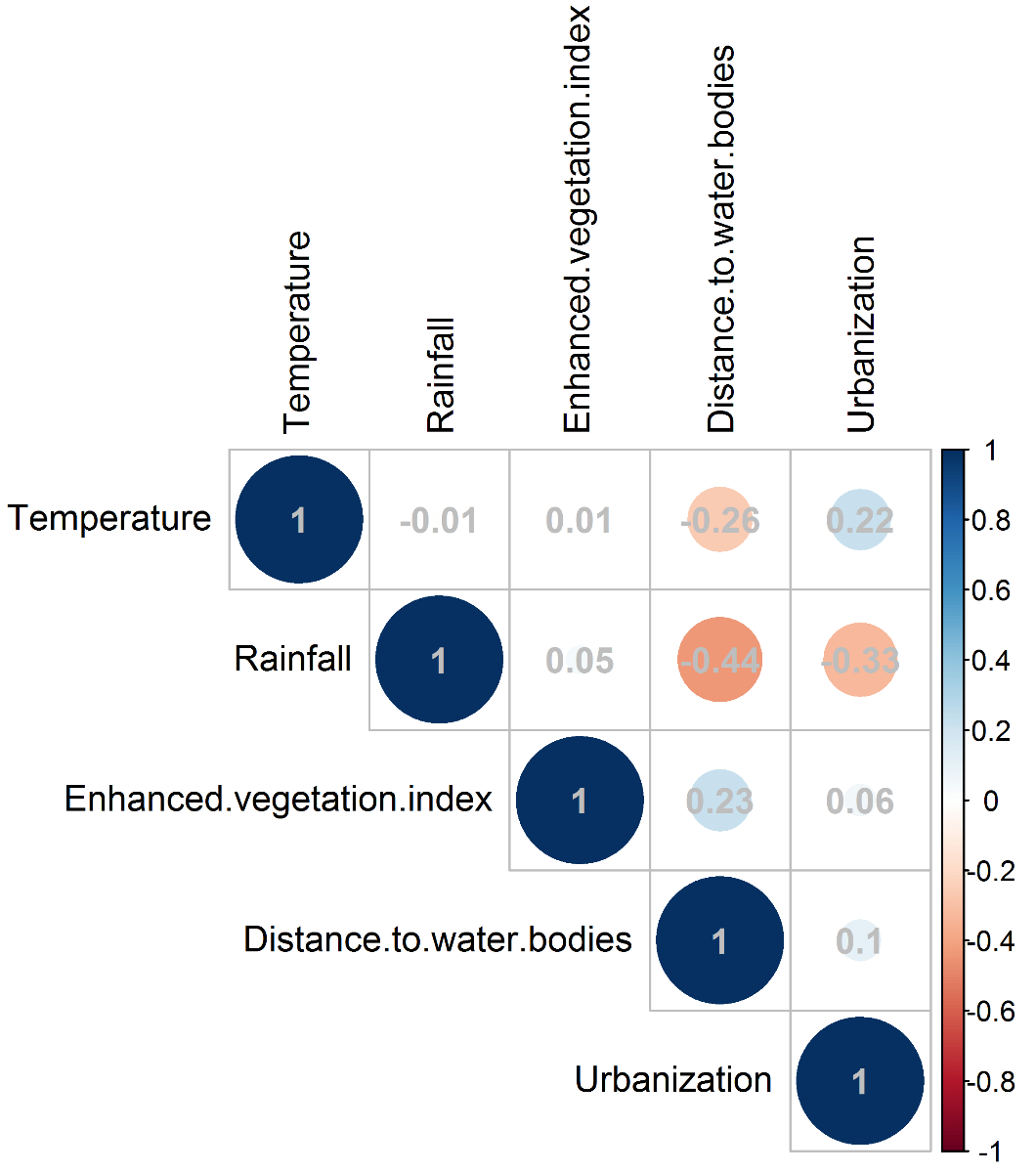
**

**Figure SI. 1:** Correlation between environmental covariates used in the joint modelling.

**Table SI.2:** Estimated regression coefficients (Odds ratio and 95% credible region (Crl)) for the posterior marginal density for the ARI*, diarrhoea and stunting among children aged 6 – 59 months in Somalia. The estimates were derived from shared component modelling and the values in bold typeface are those considered statistically significant at 95% confidence level.

| **Correlates** | | **ARI*** | | **Diarrhoea** | | | **Stunting** | |
| --- | --- | --- | --- | --- | --- | --- | --- | --- |
|  | | **Odds ratio** | **CrI** | **Odds ratio** | **CrI** | | **Odds ratio** | **CrI** |
| **Child data** | | | | | | | | |
| Vitamin A supplementation | | **0.88** | **(0.83,0.93)** | **0.92** | | **(0.86,0.98)** | 0.99 | (0.93,1.05) |
| Measles vaccination | | **0.93** | **(0.87,0.98)** | **0.91** | | **(0.85,0.97)** | 0.99 | (0.93,1.05) |
| Polio vaccination | | **0.93** | **(0.86,0.99)** | **0.87** | | **(0.81,0.93)** | **0.94** | **(0.91,0.96)** |
| Febrile Illness | | **3.04** | **(2.89,3.19)** | **2.42** | | **(2.29,2.55)** | **0.96** | **(0.93,0.98)** |
| Suspected measles | | **2.06** | **(1.87,2.27)** | **2.15** | | **(1.95,2.37)** | 1.04 | (0.93,1.17) |
| Sex of the child (Female) | | 1.02 | (0.97,1.06) | 0.96 | | (0.92,1.01) | **0.75** | **(0.71,0.78)** |
| Child age (< 12 months as reference) | 12 -< 24 months | **1.11** | **(1.05,1.18)** | **1.70** | | **(1.61,1.79)** | **1.30** | **(1.23,1.37)** |
|  | 24 – 59 months | **1.17** | **(1.08,1.27)** | **1.85** | | **(1.71,2.00)** | **2.14** | **(1.94,2.35)** |
| Age of the mother  (20-30 years as reference) | < 20 years | 0.99 | (0.88,1.11) | **1.08** | | **(0.96,1.21)** | **1.07** | **(1.03,1.10)** |
|  | 31-40 years | **0.93** | **(0.88,0.98)** | **0.93** | | **(0.88,0.99)** | **0.89** | **(0.84,0.94)** |
|  | 41-50 | **0.79** | **(0.72,0.87)** | **0.75** | | **(0.68,0.83)** | **0.93** | **(0.90,0.96)** |
|  | > 50 years | 0.71 | (0.44,1.15) | **1.43** | | **(0.92,2.21)** | 0.81 | (0.49,1.35) |
| MUAC of mother | | **0.96** | **(0.92,0.99)** | **0.98** | | **(0.97,0.98)** | 1.00 | (1.00,1.01) |
| **Household data** | |  |  |  | |  |  |  |
| Household size | | **1.02** | **(1.01,1.03)** | **1.01** | | **(1.00,1.02)** | **1.22** | **(1.20,1.23)** |
| Number of under5 | | **1.07** | **(1.04,1.10)** | **1.12** | | **(1.09,1.16)** | **1.22** | **(1.18,1.25)** |
| Female household head | | 0.97 | (0.91,1.03) | 0.96 | | (0.90,1.02) | **0.88** | **(0.83,0.94)** |
| **Food access data** | |  |  |  | |  |  |  |
| High carbohydrate foods | | **0.90** | **(0.86,0.95)** | **0.88** | | **(0.84,0.93)** | **0.82** | **(0.78,0.87)** |
| High protein foods | | **0.93** | **(0.91,0.96)** | **0.94** | | **(0.92,0.97)** | **0.76** | **(0.74,0.78)** |
| Fats | | 0.97 | (0.91,1.03) | **0.96** | | **(0.94,0.99)** | **0.92** | **(0.90,0.95)** |
| Fruits and vegetables | | **0.94** | **(0.91,0.98)** | **0.95** | | **(0.91,0.99)** | **1.07** | **(1.03,1.12)** |
| **Cluster data** | |  |  |  | |  |  |  |
| Gu (Deyr as reference) | | **0.95** | **(0.92,0.97)** | 0.95 | | (0.89,1.01) | 0.97 | (0.91,1.04) |
| Hagaa | | **0.83** | **(0.80,0.86)** | **1.05** | | **(1.01,1.09)** | 0.82 | (0.57,1.17) |
| Jillal | | **0.86** | **(0.83,0.89)** | **1.23** | | **(1.19,1.28)** | 1.07 | (0.90,1.28) |
| Enhanced Vegetation Index (EVI) | | **0.82** | **(0.80,0.85)** | **0.69** | | **(0.67,0.72)** | **0.54** | **(0.37,0.79)** |
| Rainfall | | **0.95** | **(0.91,0.98)** | **0.96** | | **(0.94,0.98)** | **0.96** | **(0.96,0.97)** |
| Temperature | | **0.94** | **(0.89,0.98)** | 0.98 | | (0.94,1.03) | **1.05** | **(1.02,1.07)** |
| Urbanization | | **0.66** | **(0.54,0.81)** | **0.82** | | **(0.71,0.94)** | **0.91** | **(0.89,0.94)** |

*Acute Respiratory Infection


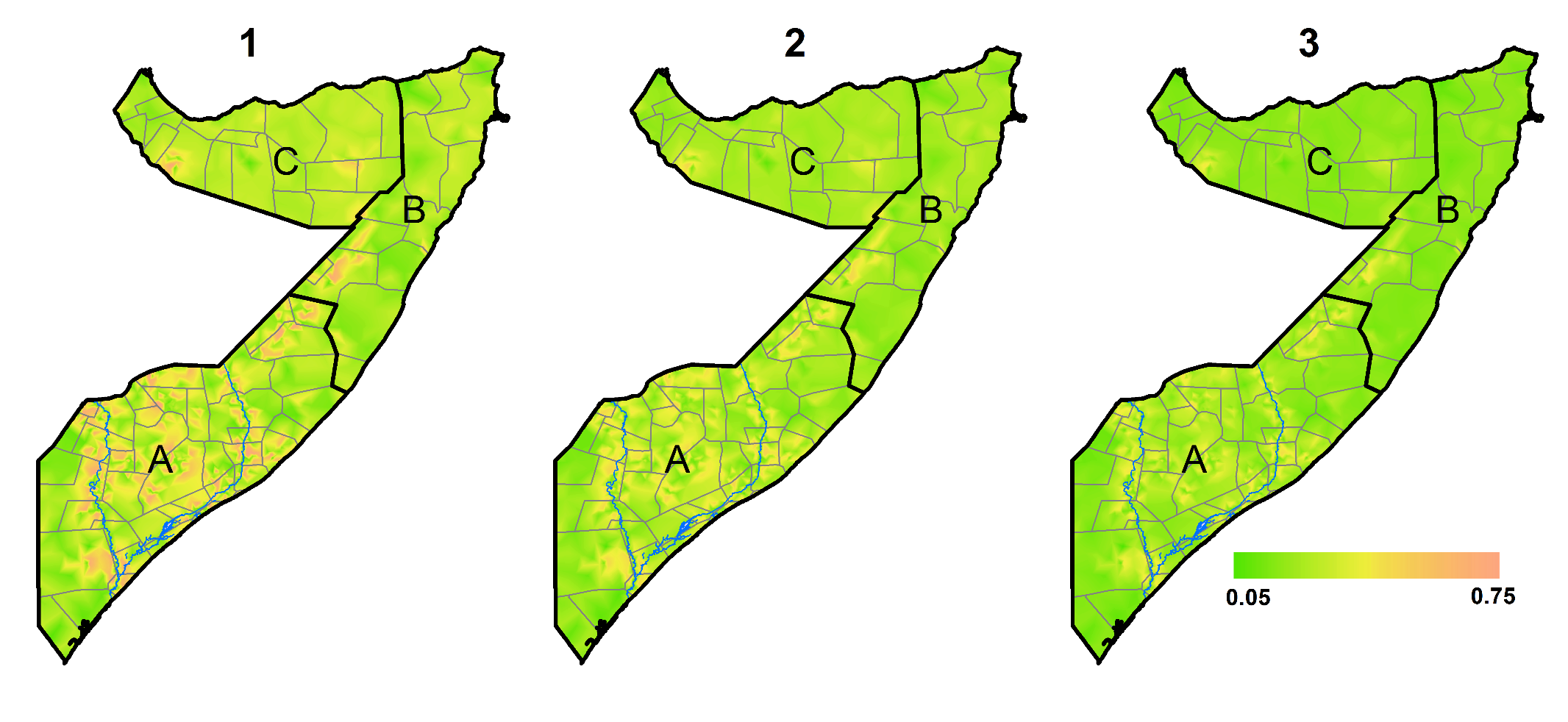


**Figure SI. 2:** Coefficients of variation at 1 x 1 km spatial resolution among children aged 6 - 59 months in Somalia. 1) Stunting and ARI, 2) Stunting and Diarrhoea 3) Diarrhoea and ARI. A=South-central zone, B=Northeast (Puntland) zone, C=Northwest (Somaliland) zone. 1=Wasting, 2=Stunting, 3=Underweight.


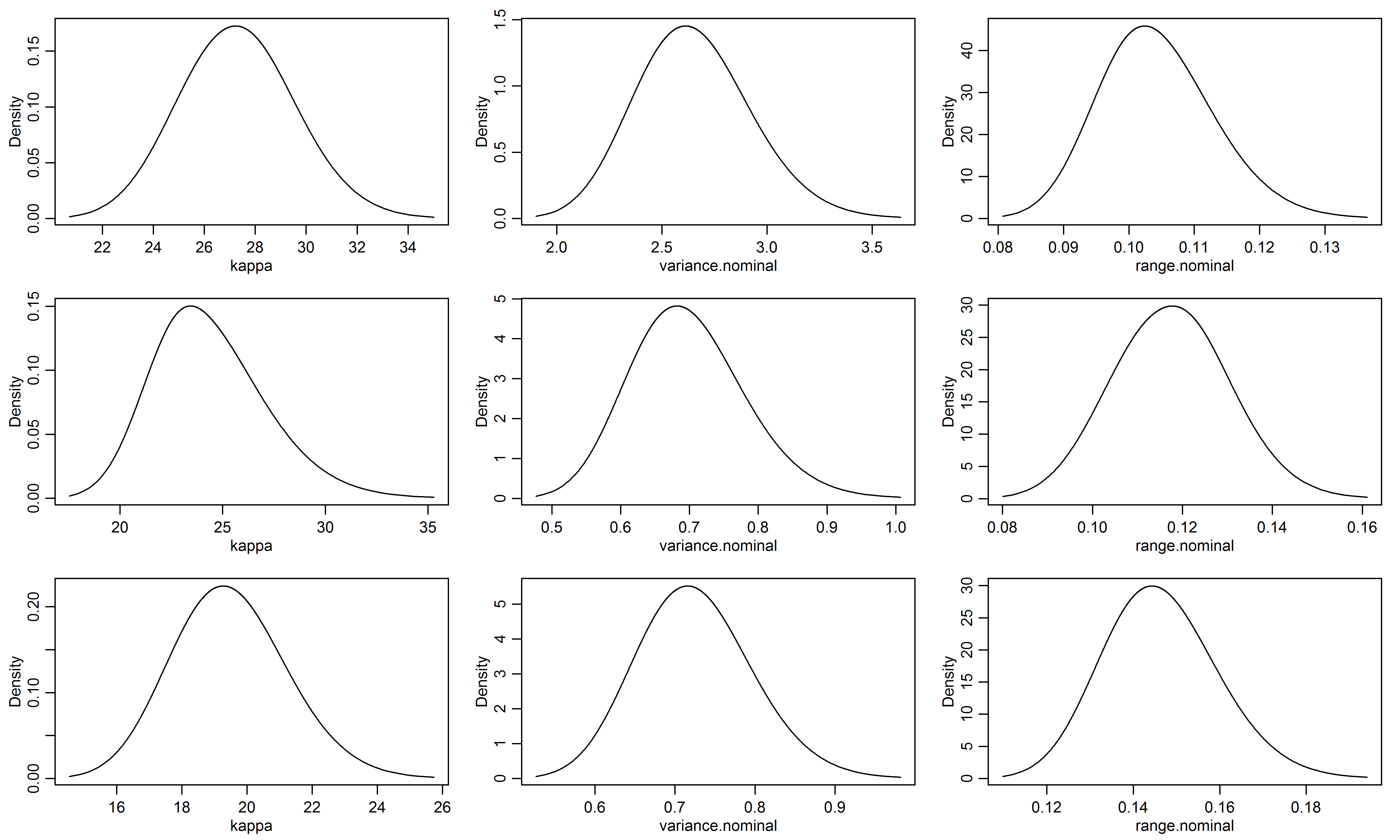


**Figure SI. 3:** Summary for the Gaussian Random Field scales which is inverse range parameters (kappa), random field marginal variances (variance.nominal) and range of the three fields for the ARI, Diarrhoea and stunting.

**References**

| 1. | Murtaugh PA. Performance of several variable-selection methods applied to real ecological data. Ecology Letters. 2009; 12(10): p. 1061-1068. |
| --- | --- |
| 2. | Scharlemann JP, Benz D, Hay SI, Purse BV, Tatem AJ, Wint GW, et al. Global data for ecology and epidemiology: a novel algorithm for temporal Fourier processing MODIS data. PLoS One. 2008; 3(1): p. e1408. |
| 3. | Schneider A, Friedl M, Potere D. A new map of global urban extent from MODIS satellite data. Environmental Research Letters. 2009; 4(4): p. 044003. |
